# Productivity Burden of Occupational Noise-Induced Hearing Loss in Australia: A Life Table Modelling Study

**DOI:** 10.3390/ijerph17134667

**Published:** 2020-06-29

**Authors:** Si Si, Kate Lewkowski, Lin Fritschi, Jane Heyworth, Danny Liew, Ian Li

**Affiliations:** 1 School of Public Health and Preventive Medicine, Monash University, Melbourne, Victoria 3000, Australia; danny.liew@monash.edu; 2 School of Public Health, Curtin University, Perth, Western Australia 6102, Australia; Katherine.Lewkowski@curtin.edu.au (K.L.); Lin.Fritschi@curtin.edu.au (L.F.); 3 School of Population and Global Health, The University of Western Australia, Perth, Western Australia 6009, Australia; Jane.Heyworth@uwa.edu.au (J.H.); Ian.Li@uwa.edu.au (I.L.)

**Keywords:** occupational noise exposure, hearing loss, economic evaluation, Australia

## Abstract

*Background:* Occupational noise-induced hearing loss (ONIHL) is one of the most common yet preventable occupational diseases. The aim of this study was to estimate the economic burden of ONIHL in the Australian working population by quantifying and monetising ONIHL—related loss of Quality Adjusted Life Years (QALY) and Productivity Adjusted Life Years (PALYs). *Methods:* We simulated the number of moderate-to-severe ONIHL by multiplying the age-specific prevalence of occupational noise exposure by the excess risks of ONIHL. Life table modelling was applied to workers with ONIHL. The QALY and PALY weights attributable to hearing loss were sourced from published data. The 2016 Gross Domestic Product per full-time equivalent worker in Australia was used to estimate the cost of productivity loss due to ONIHL. The cost due to the loss of well-being was quantified using willingness to pay thresholds derived from an Australian longitudinal study. *Results:* Under current occupational noise exposure levels in Australia, we estimated that over 80,000 male workers and over 31,000 female workers would develop ONIHL over 10 years of exposure. Following this cohort until the age of 65 years, the estimated loss of QALYs and PALYs were 62,218 and 135,561 respectively, with a projected loss of AUD 5.5 billion and AUD 21.3 billion due to well-being and productivity loss, respectively. Reducing noise exposure at work would substantially reduce the economic burden of ONIHL. *Conclusion:* ONIHL imposes substantial burden on Australian economy. Interventions to reduce occupational noise exposure are warranted.

## 1. Introduction 

Hearing loss affects over half a billion people worldwide [1] and is a leading cause of years lived with disability [2]. The two leading causes of hearing loss are ageing and noise exposure [3,4]. Studies estimating the socio-economic burden of hearing loss are limited. However, the available evidence indicates that hearing loss imposes an enormous economic burden at a societal level, primarily driven by healthcare system burden and productivity losses [5,6,7,8]. For instance, an Australian study estimated the national cost burden attributable to hearing loss to be AUD 33.3 billion in 2017. Approximately half of the cost burden was estimated to be due to financial costs, including direct health system costs and lost productivity, while the remaining half was due to the loss of well-being (quantified using disability adjusted life-years) [5].

As with most developed countries, the Australian National Standard for Occupational Noise sets the maximum daily occupational noise exposure level at an eight-hour equivalent continuous A-weighted sound pressure level (LAeq, 8h) of 85 dB [9]. One study estimated that more than 1.1 million Australian workers (20% male and 3% female workers) were exposed above this daily occupational exposure limit [10]. The prevalence of exposure differed by occupational groups, with the most exposed occupations being machine operators (65%), automotive workers (62%), construction workers (47%) and farmers (36%). Likewise, a Chinese study of occupational noise-induced hearing loss (ONIHL) in the construction industry found that the health risks differed by trades, with formwork fixers being particularly vulnerable, compared to other tradesmen such as roof bolter operators and air duct workers, even if they are present at the same construction stage and located on the same site [11]. 

ONIHL is one of the most common occupational diseases, which was estimated to account for approximately 7–21% of the burden of adult hearing loss across the world [12,13]. In Australia, more than 16,000 successful compensation claims for industrial deafness involving hearing loss were made between 2002 and 2007, which equates to more than 3000 workers annually [9]. However, it is likely that the number of successful compensation claims is an underestimation of the true number of workers affected.

Despite the high prevalence of ONIHL and substantial health and economic burden of hearing loss, studies investigating the amount and extent of ONIHL are scarce, particularly at a population level. According to Australian national standards, workplace noise levels should not exceed 85 dB. However, only workers who are deemed to be exposed above 90 dB are required to have hearing tests [9]. In addition, there is no national-level surveillance of the prevalence of occupational noise exposure, neither have there been ongoing direct measurements of existing or new cases of ONIHL in Australia [9].

Cost estimates are useful in terms of providing a policy impetus for change in practice. Specifically, evidence showing the cost burden from ONIHL and resultant cost savings could form part of the evidence base for action and change. Therefore the aims of this study included: (i) estimating the number of workers who would develop ONIHL given the current occupational noise exposure prevalence in the Australian working population; (ii) estimating the economic burden of ONIHL in the Australian working population by quantifying and monetising the related loss of quality adjusted life years (QALYs) and productivity adjusted life years (PALYs); and (iii) predicting the economic impact of workplace noise exposure reduction in the Australian working population.

## 2. Materials and Methods

We used life table modelling and decision analysis to examine the impact of ONIHL on QALYs and PALYs in Australia. Different from QALYs, which are a generic measurement of both the quantity and quality of life lived, PALYs are a measure of the time spent with reduced work productivity as a result of ill health [5,14,15]. Akin to utilities that quantify quality of life, ‘productivity indices’ represent the productivity of an individual in proportional terms, ranging from 1.0 (100% productive) to 0 (completely non-productive). 

We assumed that workers who were exposed to occupational noise levels above the daily limits and aged 30–64 years had been exposed at the same levels for 10 years, whereas for those aged 20–29 years, the same exposure level would persist for 10 years. Under these assumptions, we first calculated the excess risk (ER) of ONIHL using the current population prevalence of hearing loss from all causes (HL) and the Prevalence Ratios of ONIHL/HL assuming 10 years of occupational noise exposure. We then calculated the number of ONIHL cases under the current prevalence of occupational noise exposure in the Australian working population using the following formulas:

(1) ER=population prevalence of HL * (Prevalence Ratio−1)

(2) ONIHL=No. of exposed workers*ERs of occupational noise exposure to HL

Prevalence ratios were calculated assuming 10 years noise exposure to the risk of moderate-to-severe HL (≥40 dB) using four frequency averages of 500, 1000, 2000 and 4000 Hz via a simulation method (Tables S1–S4). The four-frequency combination was selected based on the WHO definition of hearing loss [16] which is also recommended by SafeWork Australia [9]. 

Once we had a base–case cohort of workers with ONIHL, we projected the cohort until death or age 65 years. In the meantime, we also created a counterfactual cohort of workers where no ONIHL occurred. Life tables of the two cohorts were constructed using age- and gender-specific rates of mortality for Australians aged 20–64 years [17]. The 20–64 years age range was chosen to reflect the working ages in Australia. Within each of the base–case and the counterfactual cohorts, we created separate life tables with 1-year cycles for 18 age and gender sub-cohorts, with age being stratified into nine 5-year age bands. For each sub-cohort, corresponding mortality rates were applied to people with and without ONIHL [5]. 

We calculated utilities and productivity indices in workers with and without ONIHL using data from the literature on utilities [18,19] and previously reported rates of workforce participation, absenteeism and presenteeism [5]. We applied a 5% annual discount rate to both QALYs and PALYs in the model. The differences in QALYs and PALYs between the two cohorts were used to quantify QALYs and PALYs loss due to ONIHL.

**Figure 1:**
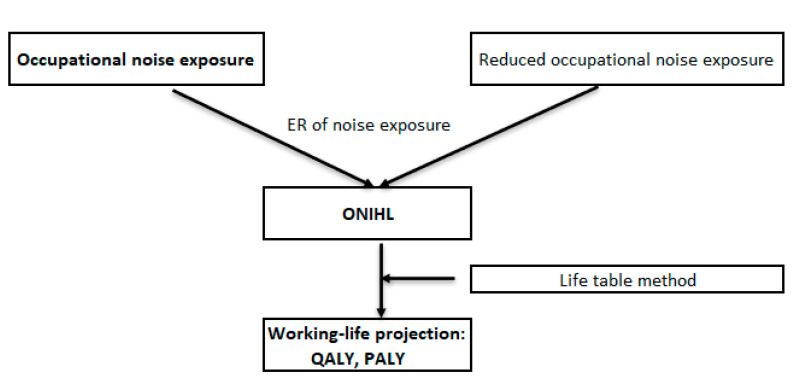
Study method flowchart (ER: excessive risk)**.**

Under an ideal scenario, the use of personal hearing protection (PHP) would reduce occupational noise exposure by 20–30 dB. However, in reality, the performance of PHP is highly dependent on the styles of PHP and more importantly, whether they have been properly fitted and are consistently used by workers. A report by the UK Health Safety and Environment estimated that under real-world conditions, less than optimal use of PHP would reduce noise exposure by 6–9 dB [20]. In line with this evidence (reducing exposure by 6-9 dB), we performed five scenario analyses that reduced the prevalence of noise exposure by 10%, 25%, 50%, 75%, and 90% in the Australian working population. Workers exposed to high-level noise (≥90 dB) were reduced to low-level exposure (85–89 dB), where workers exposed to lower-level noise (85–89 dB) were reduced to a noise level below 85 dB. The study flow chart is presented in Figure 1.

### Data Sources

Age- and gender-specific mortality rates for single-year age bands were obtained from the Australian General Record of Incidence of Mortality data for 2015 [17]. We assumed no difference in all-cause mortality among people with and without HL [5]. The age- and gender-specific prevalences of occupational noise exposure (at 85–89 dB and 90–100 dB levels) were derived from the Australian Workplace Exposure Study-Hearing (AWES-hearing, Table S5) [10]. The population prevalence of self-reported HL was derived from the 2015 Australian National Health Survey (Table S6) [21]. The prevalence ratios (PRs) of ONIHL due to noise exposure at different levels were derived from a simulation based on the Australian Standard for Acoustics (ISO1999-2013) [22]. The acoustics standard specifies algorithms/distributions for estimating the hearing threshold levels associated with age (HTLA) and noise-induced hearing threshold shift (NIHTS) based on exposure levels across the audiometric frequencies by age and gender [3]. To confer a diagnosis of hearing loss from an audiogram by the WHO diagnostic criteria, the mean of Pure Tone Averages at 500 Hz, 1000 Hz, 2000 Hz and 4000 Hz is evaluated against the diagnostic cut-offs of 25 dB, 40 dB and 60 dB for mild, moderate and severe hearing loss, respectively (Tables S1–S4). 

We calculated the utilities (quantified using QALYs) for people with ONIHL by applying a relative disutility weight (7.5% in the base-case scenario) [18] to the utilities of the Australian general population (Table S7) [19]. To further quantify the monetary value of loss of QALYs, we applied an estimate of willingness to pay (WTP) threshold derived from a nationally representative Australian longitudinal study [23]. The WTP represents the monetary value people are willing to exchange for a year of healthy/quality life. The study used a well-being valuation method, and quantified the WTP thresholds for one QALY gained at AUD 102,038 and AUD 51,805 for males and females, respectively. The study further reported the annual WTP to avoid the occurrence of long-term conditions, which they termed the long-term condition premium, at AUD 3305 and AUD 1503 for males and females, respectively.

Productivity indices were estimated using data from a Dutch survey study [24], which was previously used in an Australian and New Zealand national report of the social and economic cost of hearing loss [5,25]. According to this study, workers with/without HL had on average 12.9 and 9.3 days (out of 240 working days per annum) absent from work (absenteeism), respectively [24]. Workers with HL further incurred a 1.9% relative reduction in productivity at work (presenteeism) compared to people without HL [24]. We further derived age- and gender-specific workforce participation rates among Australians with and without HL from national statistics (Table S8) [5,26]. The cost of ONIHL-related productivity loss was estimated by assigning a unit cost for PALY, which was derived from the total Australian gross domestic product (GDP) in 2016 (AUD 1.475 trillion) [27] divided by the estimated number of full-time equivalent (FTE) Australian workers in 2016 (*n* = 9,411,998) [28]. The unit cost for PALY in 2016 was AUD 157,000 per FTE.

## 3. Results

Among Australian workers aged 20–64 years who were exposed to noise above daily limits (≥85 dB), we estimated 80,219 male and 31,517 female workers would develop ONIHL (Table 1). The estimated QALYs lost due to ONIHL were 45,526 and 16,692 for male and female workers over their working lifetime, which is equivalent to 0.57 QALYs and 0.53 QALYs per male and female ONIHL case, respectively. The estimated total WTP for QALYs lost due to ONIHL was AUD 4.6 billion and AUD 0.9 billion for male and female workers, respectively. Additionally, the long-term condition premiums were estimated to be AUD 2.5 billion and AUD 0.4 billion for male and female workers, respectively.

The total estimated lifetime productivity losses due to ONIHL were 96,871 and 38,690 PALYs for male and female workers, respectively, which is equivalent to 1.2 PALYs per male and female worker over their working life. Assuming the cost per PALY is AUD 157,000, the total cost of productivity loss attributable to ONIHL for Australian working population under current occupational noise exposure levels, was estimated to be AUD 15.2 billion and AUD 6.1 billion for male and female workers, respectively.

The impacts on QALY and PALY of noise-exposure reduction among exposed workers are summarised in Table 2. When half of the workers attained the exposure reduction target, 30,799 and 11,122 ONIHL cases would be prevented among male and female workers, respectively. This in turn translates to a total of 49,055 (35,777 + 13,278) PALYs saved and an equivalent of an AUD 7.7 billion gain in productivity. Further, the WTP for gain in well-being, as represented by the monetised values of QALYs gained, was estimated to be worth AUD 2.0 billion. A higher attainment rate (75%) in high-level noise exposure would confer productivity gains of AUD 11.6 billion in GDP (Table 2). 

## 4. Discussion

The findings of the present study illustrate the potential economic impact of ONIHL in the Australian working population. Under the current occupational noise exposure levels, among Australian workers aged 20–65 years, 111,736 workers (1.2% of the working population) were estimated to develop ONIHL, with the majority of them being males (84%). Furthermore, the losses of well-being and productivity arising from ONIHL were substantial. Specifically, a total of 62,218 QALYs and 135,561 PALYs were lost due to ONIHL. According to SafeWork Australia, there were around 3000 compensations accepted annually for occupational related hearing loss [9]. Compared to the number of compensations accepted, our analysis provided a comprehensive longitudinal estimate on both the visible and invisible socio-economic impact of ONIHL in the Australian working population. The total loss due to ONIHL was estimated to reach AUD 29.7 billion, with the majority of loss being attributed to productivity loss (72%). The magnitude of lifetime productivity loss from ONIHL found here is analogous to the annual estimate from an Australian report, in which the estimated productivity loss accounted for 81% of all costs associated with hearing loss in 2017 [5]. However, this Australian report took a snapshot of the economic burden of all-cause HL in Australian population, instead of focusing on ONIHL and its long-term impacts on loss of productivity and well-being.

In our model, higher proportions of male workers developed ONIHL compared to female workers. This accords with other studies in the literature and reflects the propensity for male workers to be employed in occupations where exposure to excessive noise is relatively common [12]. Consequently, male workers bore higher burdens of well-being and productivity loss. 

As might be expected, larger proportions of older workers were estimated to develop ONIHL. However, the proportion of younger workers who developed ONIHL was not inconsequential, particularly for male workers. For instance, it was estimated that moderate proportions of male workers in the younger age groups developed ONIHL. Specifically, we estimated 8.1% and 15.6% of ONIHL cases in male workers belonged to the 20–29 and 30–39 years age groups. As a result, the cost burden of ONIHL in these age groups was also substantial, since these costs accrue over one’s working lifetime.

As ONIHL is highly preventable, we also presented estimates on how well-being and productivity losses might be mitigated and the resultant monetary gains, when occupational noise exposure is reduced given the substantial costs associated with ONIHL that are borne by the individual (through well-being loss and reduced workforce participation), and by employers (through productivity loss in the form of absenteeism and presenteeism), addressing occupational noise exposure should be prioritised. The European Union has emphasised in the 2003 directive that collective preventive efforts including optimizing the design of work stations, equipment and working procedures are more effective than individual protection measures [29]. Our scenario analysis showed that even modest reduction of overall noise exposure (6–9 dB) is highly effective in reducing burden of ONIHL. The size of effect is proportional to attainment rate of the exposure reduction.

Several limitations and modelling assumptions warrant mention. First, our estimate of ONIHL was based on a cross-sectional survey on workplace noise exposure, the AWES-hearing study. Therefore, the sample size and the representativeness of survey sample determined the accuracy of our estimation. Second, we did not include the direct health care cost for managing hearing loss in this model because this largely depends on the diagnosis and treatment rates of hearing loss among ONIHL workers. However, the monetised values of QALY loss reflect people’s WTP for health care services to manage hearing loss. Third, in this modelling study, we assumed all workers with ONIHL were full time workers who had worked in the same occupation with the same occupational noise exposure levels for 10 years. However, we did not assume that unexposed workers changed to work in jobs with occupational noise. Fourth, as with all life table modelling exercise, we assumed the age-specific mortality remained unchanged over time. However, since hearing loss did not increase risks of all-cause mortality, it would not substantially change the relative impact of ONIHL on QALYs and PALYs. Fifth, due to the lack of evidence from Australian sources, the ONIHL related QALY decrements and PALY loss estimates were derived from the UK and the Netherlands, thus limiting the generalisability in Australian population. Finally, in this model we applied an annual discount rate of 5%. This is higher than the annual inflation rates in the past 10 years (ranging from 1.5% to 4.4%) in Australia. Thus, our model is likely to present a conservative estimate of the burden of ONIHL in the Australian working population.

## 5. Conclusions

ONIHL imposes a substantial burden on the Australian economy via productivity loss and the loss of general well-being. Interventions to reduce occupational noise exposure are warranted.

## Figures and Tables

**Table 1:** The economic burden of occupational noise-induced hearing loss (ONIHL) in Australian working population by age and sex groups.

Age Groups	# of ONIHL	QALY Loss	PALY Loss	WTP for QALYs (AUD Million)	Long-term Condition Premium (AUD Million)	Value of PALYs (AUD Million)
**Males**						
20–24 *	2126	1495	3011	$152.5	$80.3	$472.8
25–29 *	4316	2820	4807	$287.8	$152.9	$754.7
30–34	4073	4142	7890	$422.7	$223.2	$1238.7
35–39	8432	7912	15,385	$807.3	$430.8	$2415.5
40–44	5200	4391	10,238	$448.0	$241.5	$1607.4
45–49	10,374	7622	17,312	$777.7	$421.5	$2718.0
50–54	8156	4892	11,403	$499.1	$271.7	$1790.3
55–59	18,649	8097	17,748	$826.2	$448.2	$2786.4
60–64	18,893	4157	9077	$424.1	$229.0	$1425.0
Total	80,219	45,526	96,871	$4645.4	$2499.2	$15,208.8
**Females**						
20–24 *	911	574	970	$29.7	$14.0	$152.3
25–29 *	886	588	989	$30.5	$14.5	$155.3
30–34	44	45	77	$2.3	$1.1	$12.0
35–39	505	480	905	$24.9	$11.9	$142.1
40–44	4362	3740	9239	$193.8	$93.3	$1450.5
45–49	7178	5364	14,660	$277.9	$134.3	$2301.6
50–54	4356	2655	5927	$137.5	$66.8	$930.6
55–59	1576	689	1213	$35.7	$17.4	$190.5
60–64	11,699	2556	4710	$132.4	$64.8	$739.4
Total	31,517	16,692	38,690	$864.7	$418.2	$6074.3

* ONIHL occurs after 10 years.

**Table 2:** Effects of reducing high level noise exposure (>90 dB) to lower level exposure (85–89 dB) and lower level exposure to no exposure (<85 dB).

Reducing High Level Noise Exposure	# of HL Averted	QALY Gained	PALY Gained	WTP for QALYs Gained (AUD Million)	Long-term Condition Premium Gained (AUD Million)	Value of PALYs Gained (AUD Million)
				**Males**		
10% reduction	6159	3364	7156	$343.2	$184.7	$1123.4
25% reduction	15,396	8407	17,884	$857.8	$461.6	$2807.8
50% reduction	30,799	16,819	35,777	$1716.2	$923.5	$5617.0
75% reduction	46,198	25,228	53,665	$2574.2	$1385.2	$8425.4
90% reduction	55,434	30,271	64,394	$3088.8	$1662.2	$10,109.8
				**Females**		
10% reduction	2225	1160	2657	$60.1	$29.0	$417.1
25% reduction	5559	2895	6636	$150.0	$72.5	$1041.8
50% reduction	11,122	5793	13,278	$300.1	$145.1	$2084.6
75% reduction	16,687	8692	19,922	$450.3	$217.8	$3127.8
90% reduction	20,021	10,428	23,902	$540.2	$261.2	$3752.6

## Supplementary Materials

The following are available online at https://www.mdpi.com/1660-4601/17/13/4667/s1, Table S1: Quantifying the risks of hearing loss resulting from noise exposure via simulation. Table S2: Simulated Prevalence of Age-Related Hearing Loss by Gender and Age Groups; Table S3: Prevalence Ratios for Hearing Loss in Relation to Noise Exposure at 85–89 dB for 10 Years versus Age-Related Hearing Loss; Table S4: Prevalence Ratios for Hearing Loss in Relation to Noise Exposure at 90–100 dB for 10 Years versus Age-Related Hearing Loss; Table S5: Occupational noise exposure in Australian working population; Table S6: Prevalence of Age-related hearing loss and Prevalence ratios of noise-induced hearing loss; Table S7: QALY of healthy Australians by age and gender; Table S8: Australian workforce participation rates.
