# Supplementary material for: Productivity Burden of Occupational Noise-Induced Hearing Loss in Australia: A Life Table Modelling Study
Source: Int J Environ Res Public Health. 2020 Jun 29;17(13):4667. doi: 10.3390/ijerph17134667 (PMC7369732; doi:10.3390/ijerph17134667)
Supplement: Supplementary file 1 [file ijerph-17-04667-s001.pdf]

Article

Productivity Burden of Occupational Noise-Induced Hearing Loss in Australia: A Life Table Modelling Study

Si Si [1,*], Kate Lewkowski [2], Lin Fritschi [2], Jane Heyworth [3], Danny Liew [1] and Ian Li [3]

[1] School of Public Health and Preventive Medicine, Monash University, Melbourne, Victoria 3000, Australia; danny.liew@monash.edu
[2] School of Public Health, Curtin University, Perth, Western Australia 6102, Australia; Katherine.Lewkowski@curtin.edu.au (K.L.); Lin.Fritschi@curtin.edu.au (L.F.)
[3] School of Population and Global Health, The University of Western Australia, Perth, Western Australia 6009, Australia; Jane.Heyworth@uwa.edu.au (J.H.); Ian.Li@uwa.edu.au (I.L.)
* Correspondence: si.si@monash.edu

Received: 12 May 2020; Accepted: 25 June 2020; Published: 29 June 2020

Table S1. Quantifying the risks of hearing loss resulting from noise exposure via simulation.

Background

Hearing loss is determined by measuring the hearing threshold levels (HTL) in decibels over selected audiometric frequencies within the human speech range. These threshold levels are usually tested in the six octaves; 250Hz, 500Hz, 1000Hz, 2000Hz, 4000Hz and 8000Hz [1].

High levels of noise exposure, an eight-hour weighted average exceeding 85 dB, is strongly associated with increased risks of hearing loss [2].

The ISO 7029 (International Organization for Standardization, 2000) and ISO 1999–2013 specifies algorithms/distributions for estimating the hearing threshold levels associated with age (HTLA) and noise-induced hearing threshold shift (NIHTS) based on exposure levels across the audiometric frequencies by age and gender [3]. The ISO 1999-2013 standard allows audiogram patters to be simulated with or without noise exposure.

The World Health Organisation (WHO) recommends the frequency combination of 500Hz, 1000Hz, 2000Hz and 4000Hz [4]. The mean of these four HTLs is used to categorise hearing loss into mild when the pure tone average (PTA) in the better ear is between 26 and 40 decibels (dBHL), moderate as 41-60 dBHL and severe as 61–80 dBHL [4].

We aimed to estimate the prevalence ratio (PR) of Noise-Induced Hearing Loss (NIHL) via a set of simulations transferring the ISO1999-2013 into hearing loss identification by applying the WHO definition of hearing loss.

Methods

The distributions/formulas of threshold levels (including HTLA and NIHTS) at the four frequencies (500Hz, 1000Hz, 2000Hz and 4000Hz) were extracted from ISO1999-2013 [1]

$$\text{HTLA} = a\,(\text{Year-18})^2 + k\,S \qquad (1)$$

a: gender and frequency specific parameter; k: Gaussian distribution parameter; S: a distribution parameter by gender and frequency. Note: this algorithm was derived from a highly screened ontologically normal population in accordance with ISO 7029.

$$\text{NIHTS} = [u + v \lg(\theta)]\,(L_{Ex,8h})^2 - kd \qquad (2)$$

θ: Years of noise exposure; $L_{Ex,8h}$*: A-weighted noise exposure over an 8h working day; k: Gaussian distribution parameter; d: frequency specific noise exposure parameter*

Note: this formula applies to noise exposure of no less than ten years in length.

$$\text{Combined hearing threshold levels (HTL)} = \text{NIHTS} - \text{HTLA} * \text{NIHTS}/120 \qquad (3)$$

The formulas were applied to simulate the four frequency HTLs of 1000 individuals in a cohort. One random reading was allocated to an individual aged within the predefined 5-year age bands (assuming uniform distribution within each age band) from the four frequency distributions, respectively, to represent the HTL of one individual. Then the PTA was calculated for each individual. By applying the WHO criteria, we were able to determine the hearing status of this individual (i.e., no hearing loss, mild-severe hearing loss (>25dBHL) and moderate-severe hearing loss (>40dBHL)) [4]. We repeated the process 1000 times to represent 1000 individuals in a cohort. The prevalence of hearing loss was then determined in this cohort.

Occupational noise is usually measured with A-weighted decibel (dBA) over an 8-hour working day. Exposure levels of 85 dBA and 90 dBA were used as regulatory limits in developed and developing countries, respectively [4]. Noise exposure incurs cumulative damage to hearing over a long period. In this study, we apply this algorithm from ISO 1999-2013 to simulate the impact of noise exposure for at least 10 years of exposure [5,6].

Using the above method, we created 20 hypothetical sub-cohorts of the general population aged 20 to 69 years, stratified by gender and 5-year bands (thus ten different age groups), each of which comprised 1000 people. We ran simulations under three different scenarios: 1) no noise exposure (formula 1); 2) noise exposure at the level of 85-89 dBA for 10 years duration (formula 1-3); 3) noise -exposure at the level of 90-100 dBA for 10 years duration (formula 1-3) to estimate the prevalence of hearing loss for each scenario. The age and gender-specific prevalence of hearing loss under each of the three scenarios was summarised.

To represent the uncertainties of the simulated hearing loss prevalence in each sub-cohort and scenario, we repeated each cohort simulation 100 times to obtain 100 estimates of prevalence in each sub-cohort. The medians and the 2.5 and 97.5 percentiles (95% credibility intervals (CIs)) from the 100 simulations were summarised to represent the point estimate and variance of the hearing loss prevalence by age and gender. We then calculated the distribution of prevalence ratio (PR) of hearing loss associated with noise exposure by age and gender subgroups using the formula:

$$PR=\frac{\text{Prevalence}_{(\text{ARHL+NIHL})}}{\text{Prevalence}_{(\text{ARHL})}} \quad (4)$$

The median prevalence was used to derive the point estimates of PRs, whereas the upper and lower limits of 95% CIs of Age-Related Hearing Loss (ARHL)+NIHL were used to derive the 95% CIs of PRs.

**Results**
The prevalence of ARHL by age and gender are summarised in Table S2.

Table S2. Simulated Prevalence of Age-Related Hearing Loss by Gender and Age Groups.

ARHL (%)			Age Group (Years)									
			**20–24**	**25–29**	**30–34**	**35–39**	**40–44**	**45–49**	**50–54**	**55–59**	**60–64**	**65–69**
**MALES**	**Mild-severe hearing loss**	**Mean**	2.90%	2.92%	3.06%	3.16%	3.31%	3.62%	3.99%	9.69%	17.78%	27.70%
		**Median**	2.90%	2.90%	3.00%	3.20%	3.30%	3.60%	3.90%	9.60%	17.90%	27.70%
		**2.5% Percentile**	1.80%	1.95%	2.15%	2.05%	2.30%	2.45%	2.85%	7.90%	15.34%	25.20%
		**97.5% Percentile**	3.70%	3.80%	4.05%	4.11%	4.15%	4.65%	5.00%	11.55%	20.00%	29.85%
	**Moderate-severe hearing loss**	**Mean**	1.67%	1.65%	1.74%	1.79%	1.94%	1.99%	2.29%	2.60%	3.03%	3.91%
		**Median**	1.70%	1.70%	1.70%	1.80%	2.00%	2.00%	2.20%	2.60%	2.95%	3.90%
		**2.5% Percentile**	1.05%	0.90%	0.90%	0.95%	1.20%	1.20%	1.55%	1.80%	2.10%	2.85%
		**97.5% Percentile**	2.31%	2.45%	2.40%	2.55%	2.50%	2.70%	3.21%	3.55%	4.00%	4.95%
**FEMALES**	**Mild-severe hearing loss**	**Mean**	2.53%	2.47%	2.58%	2.74%	2.84%	3.12%	3.41%	3.86%	7.94%	14.70%
		**Median**	2.60%	2.50%	2.60%	2.75%	2.70%	3.10%	3.40%	3.80%	7.90%	14.60%
		**2.5% Percentile**	1.65%	1.65%	1.60%	1.85%	1.90%	2.09%	2.44%	2.65%	6.40%	13.05%
		**97.5% Percentile**	3.25%	3.15%	3.65%	3.60%	4.05%	3.90%	4.40%	4.90%	9.75%	16.70%
	**Moderate-severe hearing loss**	**Mean**	0.81%	0.79%	0.85%	0.85%	0.89%	0.97%	1.08%	1.25%	1.46%	1.85%
		**Median**	0.80%	0.80%	0.90%	0.90%	0.90%	0.90%	1.10%	1.20%	1.40%	1.80%
		**2.5% Percentile**	0.35%	0.25%	0.40%	0.40%	0.40%	0.45%	0.65%	0.70%	0.80%	1.00%
		**97.5% Percentile**	1.30%	1.50%	1.41%	1.35%	1.35%	1.50%	1.71%	1.85%	2.35%	2.71%

ARHL = age-related hearing loss.

Table S3. Prevalence Ratios for Hearing Loss in Relation to Noise Exposure at 85–89dBA for 10 Years versus Age-Related Hearing Loss.

PR * (85–89 dBA)	Males						Females					
	Mild-Severe Hearing loss			Moderate-Severe Hearing Loss			Mild-Severe Hearing Loss			Moderate-Severe Hearing Loss		
Age groups	Mean	95% LL	95% UL	Mean	95% LL	95% UL	Mean	95% LL	95% UL	Mean	95% LL	95% UL
20–24 years	1.07	0.78	1.45	1.12	0.71	1.59	1.08	0.73	1.48	1.38	0.63	2.25
25–29 years	1.10	0.78	1.48	1.06	0.76	1.53	1.16	0.78	1.54	1.38	0.81	2.25
30–34 years	1.10	0.80	1.52	1.15	0.68	1.74	1.10	0.79	1.66	1.22	0.67	2.00
35–39 years	1.06	0.75	1.42	1.11	0.67	1.58	1.09	0.76	1.53	1.22	0.56	2.28
40–44 years	1.09	0.77	1.47	1.10	0.65	1.53	1.19	0.85	1.56	1.44	0.67	2.11
45–49 years	1.11	0.86	1.52	1.15	0.75	1.73	1.13	0.85	1.48	1.44	0.83	2.50
50–54 years	2.03	1.74	2.41	1.18	0.75	1.59	1.15	0.88	1.58	1.36	0.86	2.00
55–59 years	1.60	1.43	1.86	1.12	0.83	1.60	1.86	1.51	2.30	1.42	0.83	2.25
60–64 years	1.39	1.27	1.53	1.19	0.92	1.59	1.69	1.47	1.92	1.36	0.93	2.11
65–69 years	1.27	1.18	1.39	1.64	1.32	2.00	1.43	1.29	1.60	1.44	1.00	2.11

PR = Prevalence Ratio; LL: lower limit; UL: upper limit; * PR = (prevalence of Age-Related Hearing Loss+ Noise-Induced Hearing Loss)/(prevalence of Age-Related Hearing Loss).

Table S4. Prevalence Ratios for Hearing Loss in Relation to Noise Exposure at 90–100 dBA for 10 Years versus Age-Related Hearing Loss.

**PR* (90–100 dBA)**	**Males**						**Females**					
	**Mild-Severe Hearing Loss**			**Moderate-Severe Hearing Loss**			**Mild-Severe Hearing Loss**			**Moderate-Severe Hearing Loss**		
**Age groups**	**Mean**	**95% LL**	**95% UL**	**Mean**	**95% LL**	**95% UL**	**Mean**	**95% LL**	**95% UL**	**Mean**	**95% LL**	**95% UL**
**20–24 years**	1.41	1.10	1.81	1.35	0.79	1.76	1.46	1.12	1.93	1.88	1.00	3.01
**25–29 years**	1.48	1.17	1.91	1.29	0.82	1.68	1.56	1.20	1.94	1.88	1.13	2.88
**30–34 years**	1.57	1.25	2.02	1.29	0.85	1.88	1.58	1.31	2.02	1.67	1.05	2.56
**35–39 years**	1.75	1.34	2.24	1.33	0.94	1.83	1.75	1.42	2.20	1.78	1.00	2.84
**40–44 years**	2.21	1.79	2.74	1.25	0.80	1.76	2.15	1.72	2.65	2.00	1.16	2.67
**45–49 years**	3.03	2.64	3.54	1.35	0.97	1.93	2.48	2.06	2.95	2.11	1.22	3.00
**50–54 years**	4.49	3.95	5.08	1.43	0.98	2.00	3.24	2.81	3.83	2.00	1.45	2.82
**55–59 years**	2.74	2.50	3.05	1.54	1.17	1.94	4.39	3.93	5.00	2.08	1.42	2.79
**60–64 years**	2.02	1.87	2.23	2.15	1.76	2.68	3.07	2.73	3.45	2.29	1.71	3.11
**65–69 years**	1.69	1.59	1.81	3.05	2.70	3.65	2.24	2.03	2.46	2.75	2.00	3.42

PR = Prevalence Ratio; LL = lower limit; UL = upper limit; * PR = (prevalence of Age-Related Hearing Loss+ Noise-Induced Hearing Loss)/ (prevalence of Age-Related Hearing Loss).

References

1. International Organization for Standardization. Acoustics — Estimation of noise-induced hearing loss (ISO 1999:2013). 2013. Available online: https://www.iso.org/obp/ui/fr/#iso:std:iso:1999:ed-3:v1:en (accessed on 1 June 2020).
2. National Institute for Occupational Safety and Health (NIOSH). Criteria for a Recommended Standard: Occupational Noise Exposure. Revised Criteria 1998. 1998. Available online: https://www.nonoise.org/hearing/criteria/criteria.htm (accessed on 1 June 2020).
3. International Organization for Standardization. Acoustics: Determination of Occupational Noise Exposure and Estimation of Noise-Induced Hearing Impairment. International Organization for Standardization; 1990. Available online: https://www.iso.org/standard/6759.html (accessed on 1 June 2020).
4. Concha-Barrientos, M. Occupational noise: assessing the burden of disease from work-related hearing impairment at national and local levels. Geneva: WHO; 2004. Available online: https://www.who.int/quantifying_ehimpacts/publications/9241591927/en/ (accessed on 1 June 2020).
5. Thorne, P.; Ameratunga, S.; Dodd, G.; Purdy, S.; Reid, N.; Williams, W. Best practice in noise-induced hearing loss management and prevention: A review of literature, practices and policies for the New Zealand context. 2006. Available online: https://dspace.nal.gov.au/xmlui/bitstream/handle/123456789/481/Best%20practice%20in%20noise%20-%20induced%20hearing%20loss%20management%20and%20prevention.pdf?sequence=1&isAllowed=y (accessed on 1 June 2020).
6. Hallberg, L. Occupational hearing loss: coping and family life. *Scand. Audiol. Suppl.* **1996**, *43*, 25–33.

Parameters Used in the Economic Modelling

Table S5. Occupational noise exposure in Australian working population.

		20–24	25–29	30–34	35–39	40–44	45–49	50–54	55–59	60–64
MALE	**pop_exposed (85–90)**	**54082**	**66310**	**51650**	**52999**	**53011**	**59948**	**45719**	**40130**	**23923**
	95%LL (85–90)	45021	57592	43505	47169	46108	52470	38855	35237	19997
	95%UL (85–90)	63143	75027	59796	58828	59914	67426	52582	45021	27850
	**pop_exposed (>90)**	**52797**	**88127**	**69405**	**92213**	**68239**	**66607**	**39925**	**57533**	**27657**
	95%LL (>90)	25840	51638	38404	61642	42284	41848	22778	37350	15149
	95%UL (>90)	79754	124616	100406	122783	94194	91365	57071	77716	40165
FEMALE	**pop_exposed (85–90)**	**17589**	**4355**	**1340**	**1720**	**12903**	**4218**	**5770**	**4496**	**981**
	95%LL (85–90)	3823	0	0	0	7975	2646	3412	0	625
	95%UL (85–90)	27443	7822	6079	3737	17831	5789	8127	9324	1339
	**pop_exposed (>90)**	**4562**	**5001**	**0**	**2290**	**13083**	**18464**	**11487**	**1056**	**17175**
	95%LL (>90)	0	0	0	0	541	3378	722	0	3623
	95%UL (>90)	13035	14560	0	8573	25625	33551	22253	4824	30726

LL = lower limit; UL = upper limit; Data source: Lewkowski, K.H.J.; Li, I.W.; Williams, W.; McCausland, K.; Gray, C.; Ytterstad, E.; Glass, D.; Fuente, A.; Si, S.; Florath, I.; Fritschi, L. Exposure to noise and ototoxic chemicals in the Australian workforce. *J. Occup. Med.* **2019**, *76*, 341–348.

.

Table 6. Prevalence of Age-related hearing loss & Prevalence ratios of noise-induced hearing loss.

**Self-Reported HL (%) ***		20–24	25–29	30–34	35–39	40–44	45–49	50–54	55–59	60–64
**Males**		8.51%	14.61%	14.61%	23.25%	23.25%	32.11%	32.11%	51.97%	51.97%
**Females**		8.51%	14.61%	14.61%	23.25%	23.25%	32.11%	32.11%	51.97%	51.97%
PR of noise-induced hearing loss at exposure level of 85–89 dBA for 10 years										
**Males**	**MEAN**	1.12	1.06	1.15	1.11	1.1	1.15	1.18	1.12	1.19
	95% LL	0.71	0.76	0.68	0.67	0.65	0.75	0.75	0.83	0.92
	95% UL	1.59	1.53	1.74	1.58	1.53	1.73	1.59	1.6	1.59
**Females**	**MEAN**	1.38	1.38	1.22	1.22	1.44	1.44	1.36	1.42	1.36
	95% LL	0.63	0.81	0.67	0.56	0.67	0.83	0.86	0.83	0.93
	95% UL	2.25	2.25	2	2.28	2.11	2.5	2	2.25	2.11
PR of noise-induced hearing loss at exposure level of 90-100 dBA for 10 years										
**Males**	**MEAN**	1.35	1.29	1.29	1.33	1.25	1.35	1.43	1.54	2.15
	95% LL	0.79	0.82	0.85	0.94	0.8	0.97	0.98	1.17	1.76
	95% UL	1.76	1.68	1.88	1.83	1.76	1.93	2	1.94	2.68
**Females**	**MEAN**	1.88	1.88	1.67	1.78	2	2.11	2	2.08	2.29
	95% LL	1	1.13	1.05	1	1.16	1.22	1.45	1.42	1.71
	95% UL	3.01	2.88	2.56	2.84	2.67	3	2.82	2.79	3.11

Data source: Australian Bureau of Statistics. National Health Survey 2014–2015. 2015. Available online: https://www.abs.gov.au/AUSSTATS/abs@.nsf/DetailsPage/4364.0.55.0012014-15?OpenDocument (accessed on 1 June 2020).

Table S7. QALY of healthy Australians by age and gender.

Age	Males	Females
15	0.88	0.87
25	0.88	0.84
35	0.84	0.84
45	0.81	0.81
55	0.79	0.8
65	0.8	0.79
75	0.79	0.76

Data source: Hawthorne, G.; Korn, S.; Richardson, J. Population norms for the AQoL derived from the 2007 Australian National Survey of Mental Health and Wellbeing. Australian and New Zealand *Int. J. Public Health.* **2013**, *37*, 7–16.

Table 8. Australian work force participation rates.

Age	Females		Males	
	HL-	HL+	HL-	HL+
15–19	48%	35%	42%	1%
20–24	74%	33%	74%	50%
25–29	77%	68%	87%	51%
30–34	76%	67%	90%	74%
35–39	75%	76%	89%	92%
40–44	78%	75%	92%	77%
45–49	80%	59%	89%	80%
50–54	77%	57%	88%	74%
55–59	70%	62%	81%	70%
60–64	49%	39%	65%	53%

Data source: Australian Bureau of Statistics. Disability, Ageing and Carers, Australia: Summary of Findings. Canberra: Australian Bureau of Statistics; 2015. Available online: https://www.abs.gov.au/AUSSTATS/abs@.nsf/DetailsPage/4430.02018?OpenDocument (accessed on 1 June 2020).
